# Improving Machine Vision Using Human Perceptual Representations: The Case of Planar Reflection Symmetry for Object Classification

**DOI:** 10.1109/TPAMI.2020.3008107

**Published:** 2020-07-09

**Authors:** RT Pramod, SP Arun

**Affiliations:** Center for Neuroscience and the Department of Electrical Communication Engineering, Indian Institute of Science, Bangalore, India, 560012

**Keywords:** Object Recognition, Computational models of Vision, Perception and Psychophysics

## Abstract

Achieving human-like visual abilities is a holy grail for machine vision, yet precisely how insights from human vision can improve machines has remained unclear. Here, we demonstrate two key conceptual advances: First, we show that most machine vision models are systematically different from human object perception. To do so, we collected a large dataset of perceptual distances between isolated objects in humans and asked whether these perceptual data can be predicted by many common machine vision algorithms. We found that while the best algorithms explain ∼70% of the variance in the perceptual data, all the algorithms we tested make systematic errors on several types of objects. In particular, machine algorithms underestimated distances between symmetric objects compared to human perception. Second, we show that fixing these systematic biases can lead to substantial gains in classification performance. In particular, augmenting a state-of-the-art convolutional neural network with planar/reflection symmetry scores along multiple axes produced significant improvements in classification accuracy (1-10%) across categories. These results show that machine vision can be improved by discovering and fixing systematic differences from human vision.

## 1 Introduction

When [the Master] makes a mistake, he realizes it.

Having realized it, he admits it.

Having admitted it, he corrects it.

Tao Te Ching, v61 [1]

Convolutional neural networks (CNNs) have revolutionized computer vision with their impressive performance on object recognition [2], [3], [4], [5]. Their performance, although impressive compared to other machine algorithms, is still inferior to humans [6]. The performance gap between machines and humans is even more striking when one compares top-1 accuracy: for instance, the accuracy for finding cars in natural scenes is ∼80% for CNNs and 93% for humans [7]. Can we use insights from human vision to bridge this performance gap? While it is relatively straightforward to identify objects and images on which humans perform better than machines [6], using these observations to improve machines is non-trivial for several reasons. First, better performance could be due to better classifiers or image features. Second, these observations tend to be class-specific and rarely point to generic image properties that should be included during training. In the visual cortex, neural responses are modulated by task demands but feature selectivity remains unaltered [8]. Third, classification accuracy is a discrete measure that is insensitive to fine-grained variations across objects within a given object class. Finally, although abstract principles such as Gestalt have been extensively characterized in humans [9], [10], it is unclear how they contribute to recognition, and also unclear how to determine if they are present in machine vision algorithms.

A simpler alternative therefore would be to measure distances between objects in feature space. In machines, this can be done by calculating metric distances between feature vectors. In humans, these distances can be measured experimentally in behavior [11], [12], [13] or in specific brain regions [14], [15].

Here we compared object representations in human perception with machine algorithms, discovered image properties that are systematically biased in machines, and improved state-of-the-art machine algorithms by augmenting them with these discovered properties. To measure feature representations in humans, we measured perceptual dissimilarity using visual search. Visual search is an extremely intuitive task where performance can be measured objectively, and the time taken to find the search target can be taken as an index of similarity. The reciprocal of search time serves as a useful measure of dissimilarity that behaves like a distance metric [12] and combines linearly across both object properties [16], [17], [18] as well as top-down factors [19]. Further, asymmetries and set size systematically modulate search but do not alter the rank ordering of search difficulty [12], [16]. Although subjects might make multiple eye movements during search, their search dissimilarity is predictable from the first few hundred milliseconds of neural activity in the higher visual areas, suggesting that search dissimilarity is driven largely by feedforward processing [20], [21], [22]. Finally, we note that while it is appealing to measure perceptual dissimilarity on natural scenes, interpreting this data can be complicated because the dissimilarity could be based on looking at multiple objects in a scene. Therefore we used objects isolated from their background in the human experiments to probe their underlying representation.

Using this approach, we measured a large set of perceptual dissimilarities and compared the ability of many common machine algorithms to explain these data. This analysis revealed several systematic biases between machines and human perception. The most notable bias was that symmetric objects were more distinct in human perception compared to most machine algorithms. Symmetry is an important property in our perception [9], [10], [23] that we detect far better than machine algorithms [24]. Symmetry detection in an image is a challenging problem that has been studied extensively [24], [25], [26] including more recently using neural networks [27], [28], [29], [30], [31]. Recent studies have suggested a role for local ribbon symmetry in contours in scene categorization [32]. Despite these insights it is not clear whether detecting symmetry is useful for large-scale object recognition, and whether it is already learned by CNNs over the course of their training. We therefore augmented CNNs with symmetry features, and confirmed that this indeed resulted in significant improvements in performance. Our approach is validated by the fact that we obtained significant improvements on natural scenes despite discovering this bias using isolated objects. Finally, we show that CNNs represent symmetry differently because the units that contribute the most to classification have weaker symmetry bias and are tuned to high spatial frequencies.

### 1.1 Background

Below we review previous work in comparing machine and human vision. Machines and humans have traditionally been compared using their performance on many vision tasks from recognition [2], [4], [5], [6] to segmentation [33]. However comparing overall task performance is problematic for inference because any difference could be due to the underlying features or due to the underlying decision process that produces the eventual behavioral response. More recently, object representations have been characterized using human behavior [11], [12], [13], [34], [35] and in distinct brain regions [14], [15]. There are two broad findings from these studies: First, object representations in early visual cortex are explained by Gabor filters [36] or the Gabor-like representations found in early layers of CNNs [37]. Second, object representations in higher visual areas in both humans (using fMRI/MEG) and monkeys (using single neuron activity) are explained better using SIFT [14] and HMAX models [15], and more recently, by later layers of CNNs optimized for object classification [15], [38], [39], [40]. The similarity between brains and CNNs predicts similar, not inferior performance for CNNs compared to humans. Thus these results do not explain the performance gap between CNNs and humans.

This apparent contradiction could have arisen for two reasons: First, most of these comparisons are based on natural objects containing many features. This could have produced a large correlation between object distances even if the underlying features are entirely different. Second, there may be systematic differences between machine vision algorithms and brains for some types of images but not others. For example, images of cars or images with straight lines could show similar representations in both human perception and computer vision models whereas images of faces or images with curved lines could show systematic differences in representations between humans and machines. To the best of our knowledge, these issues have never been investigated. Even if systematic differences are identified [41], it is plausible but by no means certain that incorporating these differences will lead to tangible gains in performance [42].

Can we use brain data to improve machine vision? There is extensive evidence that augmenting images with virtually any human annotation can yield significant improvements, but these studies typically assume human-assisted situations where manual annotation is always available [43], [44]. But can human annotations be automated and then used to improve machine vision in novel images lacking annotation? There has been surprisingly little work to address this question. A recent study has augmented CNNs with human-derived contextual expectations to show improved performance [45]. Another recent study has shown that using brain data to constrain machine learning can lead to improved performance [46]. Yet another study uses a method called *Data Distillation* to generate annotations on unlabeled datasets and increase the size of the training data in order to improve model performance on various vision tasks [47]. These studies show that human-derived data can improve machine vision but do not reveal any systematic biases in machine vision that may have been lacking in the first place.

### 1.2 Overview and contributions of this study

There are several novel aspects to this study. First, we have shown that perceptual similarity between objects in humans can be systematically measured and modeled using computer vision algorithms. To this end we are making publicly available a large dataset-the IISc-Dissimilarity between Isolated Objects Dataset-containing 26,675 perceptual distances between 2,801 objects measured from 269 human subjects. Second, we show that nearly all computer vision models tested show systematic biases from human perception. In particular we show that symmetric objects are more distinct in perception compared to all computational models. Third, we show that augmenting state-of-the-art CNNs with symmetry features leads to tangible gains in performance. This finding is non-trivial because the systematic biases in humans may be present to serve visual functions other than classification. It is also nontrivial because state-of-the-art CNNs are already optimized for existing datasets and therefore augmenting them may not improve their performance. These results are a proof-of-principle of this approach: that fixing systematic differences between machine and human vision can lead to concrete improvements in machine vision. Some of these results have been presented previously [34], although we have expanded upon this work considerably.

In Section 2, we describe the collection and validation of the perceptual data and comparison with computational models. In Section 3, we describe how CNNs can be improved by including symmetry features. In Section 4, we analyze CNN unit activations to elucidate why they show a bias in representing symmetric objects, and discuss how CNNs could be trained to overcome this bias.

## 2 Comparing Machine And Human Vision

Here we collected a large dataset of perceived dissimilarity measurements between pairs of images and tested a large number of computational models for their ability to explain these data. These analyses revealed several systematic biases between all computational models and perception.

### 2.1 Dissimilarity measurements in humans

**Fig. 1:**
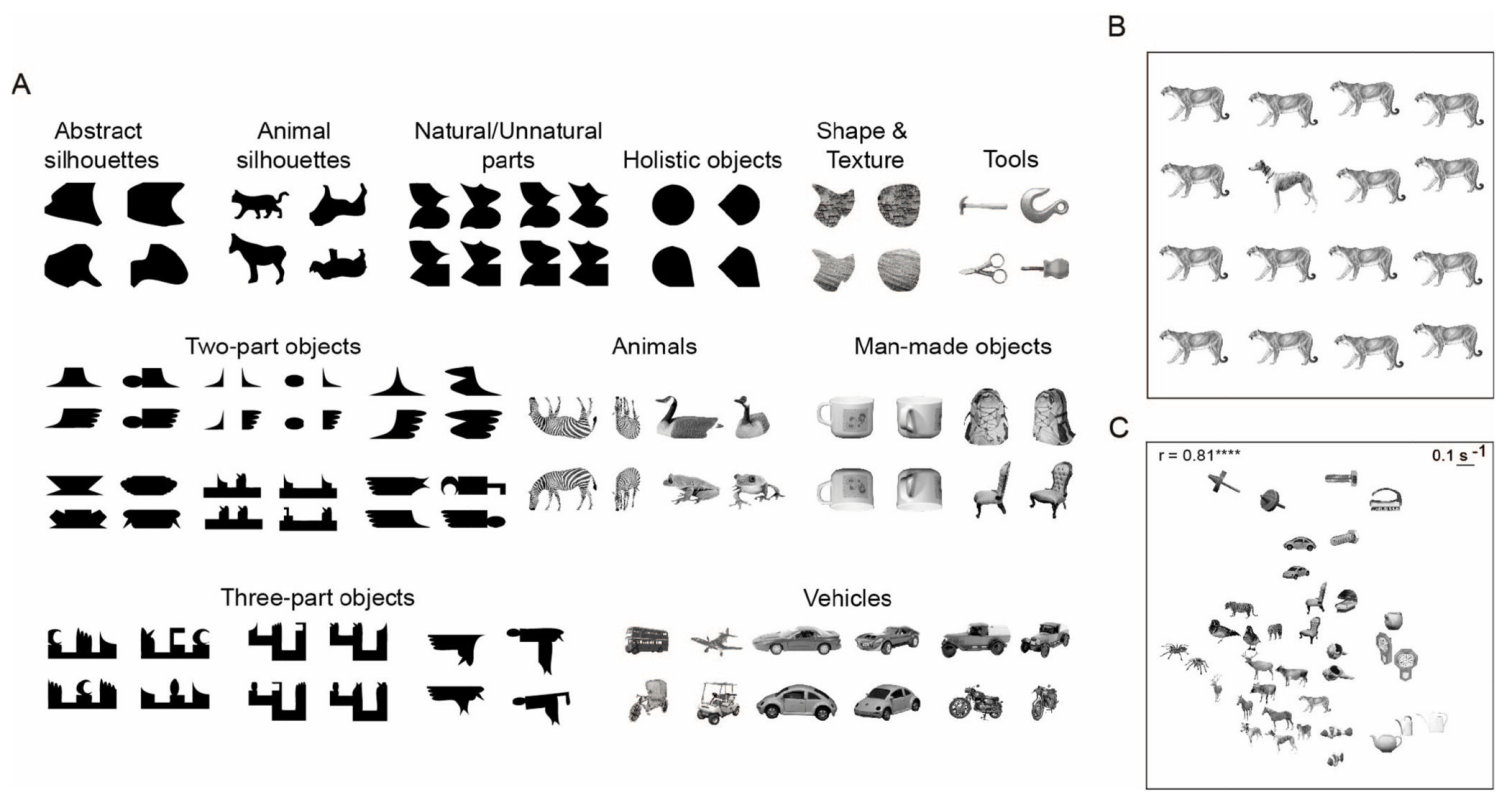
Stimuli and Experiment. (A) Example objects used in the study for measuring perceived dissimilarities in humans; (B) Example 4x4 visual search array with one oddball target (dog) amongst multiple instances of the distractor (cougar); (C) 2D embedding of measured distances between a set of natural images, as obtained using Multidimensional scaling (MDS). The r-value indicates the agreement between search distances and the embedded distances (**** is p < 0.00005).

To compare object representations in humans and machines, we collected a dataset of 2,801 objects containing natural objects and silhouettes (See Figure 1A for example objects). The natural objects were drawn from various natural object categories like animals, vehicles and tools. For some natural objects, there were two views: a profile (sideways) view and an oblique view created by in-depth rotation of the profile view. The silhouette shapes also varied in complexity from simple to complex, and in familiarity from abstract to familiar. A subset of these silhouette shapes were created by combining 7 possible parts on either end of a stem to get a total of 49 objects (Figure 5A). The set of 2,801 objects were presented across 32 separate experiments each typically with at least 8 subjects. In each experiment, we measured perceived dissimilarity between pairs of objects using a visual search paradigm as given below. In total, we measured perceived dissimilarity for 26,675 pairs of objects across 269 human subjects.

All participants were aged 20-30 years, had normal or corrected-to-normal vision, naive to the purpose of the experiments and gave written informed consent to an experimental protocol approved by the Institutional Human Ethics Committee of the Indian Institute of Science. All experiments were conducted in a darkened room. Subjects were seated approximately 60 cm from a computer monitor controlled by custom programs written using Psychtoolbox [48] in MATLAB. At the beginning of each trial, a fixation cross appeared at the center of the screen for 500 ms. Following this an array of 16 items appeared in a 4x4 grid, which contained one oddball image and 15 identical distractor images (e.g. see Figure 1B). In most experiments, the search array measured 21° × 21° with the items measuring 3° along the longer dimension. The location of the distracter was randomly chosen with equal probability of occurance in all 16 locations. We jittered the position of items in the array to prevent alignment cues from driving the search. Subjects were instructed to respond as quickly and as accurately as possible to indicate the side on which the oddball target was present using a pre-specified key press (Z for left and M for right, on a QWERTY keyboard). To facilitate this, all search arrays had a red vertical line running down the middle of the display. The search array stayed on for 10 s or until the subject responded, whichever was earlier. All aborted or incorrect trials were repeated at a random time-point later in the task. Depending on the experiment, subjects performed between 2-8 correct trials for each pair of objects. We recorded the response time for each trial.

For each search, we took the reciprocal of the average search time as an estimate of perceived dissimilarity between the target and distractor. This measure behaves like a mathematical distance metric [12], shows linear summation across multiple features [17], [18] and correlates with measures of subjective dissimilarity [17].

#### Search asymmetry

It has been observed previously that, for some object pairs, search can be asymmetric. For example, searching for Q among O’s is significantly faster than searching for O among Q’s [49]. We therefore analysed our data for the presence of asymmetries. To this end, we selected all object pairs with at least 8 trials (n = 200) and for each pair, we performed an analysis of variance (ANOVA) on search reaction times with subject and asymmetry (each item as target) as factors. Across the 200 pairs, 27 pairs (13.5%) showed a significant main effect of asymmetry after correcting for multiple comparisons (p < 0.05, Bonferroni corrected). Thus, search asymmetries are relatively rare in our dataset.

#### Dataset consistency

Since the complete dataset was collected from many human subjects, we were concerned that the measurements may not be representative of the perceptual distances within any given subject. However this is unlikely for the following two reasons: First, comparing the average dissimilarity between two random halves of the subjects yielded an extremely high correlation (r = 0.84, p < 0.00005; Pearson’s product-moment correlation coefficient). Second, in a separate experiment, we measured perceptual distances for a random subset of 400 image pairs from the full dataset in four human subjects. These perceptual distances were strongly correlated with the original dataset (r = 0.80, p < 0.00005; Pearson’s product-moment correlation coefficient). Further, the distributions of perceived distances measured in the main and control experiments were not significantly different (median perceptual distances: 0.98*s* ^–1^ for the control experiment and 0.94*s* ^–1^ for the main experiment; p = 0.9 for a ranksum test on perceived distances).

### 2.2 Computer vision models

We tested a total of 23 popular computer vision models. We grouped these models roughly into five categories for ease of exposition: pixel-based, boundary-based, featurebased, statistical and biologically-inspired network models. For most models, we extracted the feature vector for each image and calculated the Euclidean (or city-block) distance between the feature vectors. For some models (like, Curvature Scale Space model) which were specified in terms of a distance metric rather than a feature vector, we computed the pairwise distances directly. All images in the dataset were scaled to a square frame of 140 pixels (or modelspecific size esp. for convolutional neural networks) before giving as input to each model. Each model has been described in detail previously [34].

### 2.3 Model evaluation

Because some of the computer vision models we tested are already optimized for classification (e.g. CNNs), we evaluated models in two ways. First, we calculated the direct correlation between model distances and observed perceptual distances. Second, we fit each model to the perceptual data by weighting its features to obtain the best match to the data. We used a standard cross-validation approach where the model was trained on 80% of the data and tested on the remaining 20%.

To equate predictive power across all models, we performed dimensionality reduction using Principal Component Analysis (PCA) and reduced each model’s feature representation into a 100-dimensional feature vector per image. We then asked if a weighted sum of distances along these 100 principal components could explain the observed perceptual data better. Specifically, if *x*_1_ = [*x*_1,1_ *x*_1,2_ *x*_1,3_ … *x*_1,100_] and *x*_2_ = [*x*_2,1_ *x*_2,2_ *x*_2,3_, … *x*_2,100_] are the 100-dimensional feature vectors corresponding to two images, then our model predicts the observed distance y_12_ between these two images to be:

(1) $$y_{12}=w_{1}\left|x_{1,1}-x_{2,1}\right|+w_{2}\left|x_{1,2}-x_{2,2}\right|+\ldots +w_{100}\left|x_{1,100}-x_{2,100}\right|$$

where *w*_1_, *w*_2_ etc represent the contribution of that particular principal component to the overall perceptual distance.

In addition to the 23 individual models, we asked whether combining all models would yield better predictions of the observed perceptual data. To this end, we tested two combined models. In the first combined model (hereafter, *comb1*), we concatenated z-scored feature vectors from 15 individual models (out of the 23 models considered, we excluded 4 network based models in favor of VGG-16 as it on its own yielded better fit to the observed data; among the other 4 excluded models, SSIM does not have explicit feature representation, CSS and GB have very few features and the V1 model had too many features to perform PCA). We then further reduced the concatenated feature representation to 100 dimensions using PCA. We repeated the weighted summation and cross-validation procedures as described above to characterize the model performance.

In the second combined model (hereafter, *comb2*), we predicted perceptual distances as a weighted sum of individual model distances. Specifically, we solved a matrix equation of the form *y* = *Xb*, where *y* is a 26,675 × 1 vector containing observed distances, *X* is a 26,675 × 23 matrix containing (feature unweighted) distances predicted by each of the 23 models and *b* is an unknown 23 x 1 weight vector representing the relative contribution of each model to the observed distances.

#### 2.3.1 Evaluating model quality-of-fit

We estimated the amount of explainable variance or reliability of the observed data by calculating the splithalf correlation. Specifically, we separated the subjects into two random groups and calculated the perceptual distances separately. We then computed the correlation between perceptual distances for these two groups and reasoned that the degree to which these two random groups are correlated would be the upper limit for any model fit. However, split-half correlation computed this way cannot be used directly as it may underestimate the true reliability of the data. This is because split-half correlation is based on comparing two randomly selected halves of the data whereas models are trained on the entire dataset. We therefore corrected the split-half correlation using the Spearman-Brown formula, given by $r_{c}=\frac{2r}{\left(1+r\right)}$, where *r* is the split-half correlation and *r_c_* is the corrected correlation. We calculated a composite measure of model performance as the squared ratio between model correlation and corrected split-half correlation.

(2) $$\text{\% variance explained }={\left(\frac{r_{m}}{r_{c}}\right)}^{2}$$

where *r_m_* is the model correlation with the observed data. All correlation coefficients reported in this study are Pearson’s product-moment correlation coefficients.

#### 2.3.2 Strength of symmetry

Throughout, by ‘symmetry’ we mean the specific case of planar reflection symmetry about any axis in the image plane [50]. To quantify the strength of symmetry of an object, we computed the degree to which two halves of the object are mirror images of each other. Specifically, the pixel-wise difference between two halves of a symmetric object, mirrored about the axis of symmetry, will be zero. Thus, we defined the strength of symmetry about the vertical axis for an object *A* as:

(3) $$S_{v}=1-\frac{\sum \text{abs}(A-\text{flipv}(A))}{\sum \text{abs}(A+\text{flipv}(A))}$$

Where flipv(*A*) represents the object mirrored about the vertical axis, and abs() is the absolute value, and the summation is taken over all pixels. This strength of symmetry measure is 0 when the object and its vertical mirror reflection do not overlap at all, and is 1 when the object and its vertical mirror reflection are identical in every pixel (i.e. when the object is symmetric). In addition to this, we also calculated strength of symmetry about the horizontal axis (*S_h_*) in a similar manner. For each pair of objects, we calculated the strength of symmetry about vertical axis averaged over both objects, and similarly strength of symmetry about horizontal axis averaged over both objects. The overall strength of symmetry for a given pair was computed as the larger of the vertical and horizontal symmetry measures. This way of measuring symmetry is appropriate in our case because all objects were centered in the image and hence had their axes of symmetry passing through the center of the image. Further, we did not account for skew-symmetry as only few natural objects in our dataset showed out of picture plane rotations.

### 2.4 Results

#### 2.4.1 Comparing perception and computer vision models

We measured perceived dissimilarity for 26,675 pairs of objects taken from 2,801 objects across 269 human subjects using a visual search paradigm (See Figure 1A for example objects and Figure 1B for an example visual search array). We only tested a subset of all object pairs due to experimental constraints as well as to avoid testing completely dissimilar objects that would yield only extreme values in the range. Specifically, the reciprocal of search reaction time was used as a measure of perceived dissimilarity [12].

Subjects were highly consistent in their performance, as evidenced by a strong correlation between the distances measured from two halves of subjects across all object pairs (split-half correlation: r = 0.81, p < 0.0005). This degree of consistency is striking, particularly considering that eye movement patterns, attentional engagement could have varied across subjects, and target eccentricity and item spacing were not held constant across experiments.

To visualize these dissimilarities, we used Multidimensional Scaling (MDS) to embed objects into two dimensions such that their distances best approximated the observed distances (Figure 1C). In the resulting plot, nearby objects represent hard searches. Interestingly, profile and oblique views of natural objects are close together, indicative of viewpoint invariance in human perception. It can also be seen that animate objects form a cluster indicative of their shared features.

Next, we asked whether distances between objects in computational models (without fitting to the data) are correlated with perceptual data. For each model, we took the feature vectors that are typically used for classification, and calculated distances between objects using the Euclidean distance between the corresponding feature vectors. As described in the previous section, we quantified model performance (or % variance explained) as the squared ratio between model correlation and corrected split-half correlation. All computational models showed a significant positive correlation with perceptual data with the VGG-16 model achieving the best performance (r = 0.68, p < 0.00005). This model explained 55.1% of the explainable variance in the data. Interestingly, GoogLeNet did not do better than VGG-16 on this dataset even though it achieves significantly better classification results on the ImageNet dataset [2], [4], [5]. Further, when we allowed models to fit to the perceptual data by re-weighting their features (100-dimensional feature vectors from PCA, see previous section), most models improved in their performance. Still, VGG-16 was the best model and explained 62.6% of the explainable variance (r = 0.72, p < 0.00005). Further, the observed trend in model fits remained similar when we used feature vectors with 50 dimensions instead of 100 dimensions.

**Fig. 2:**
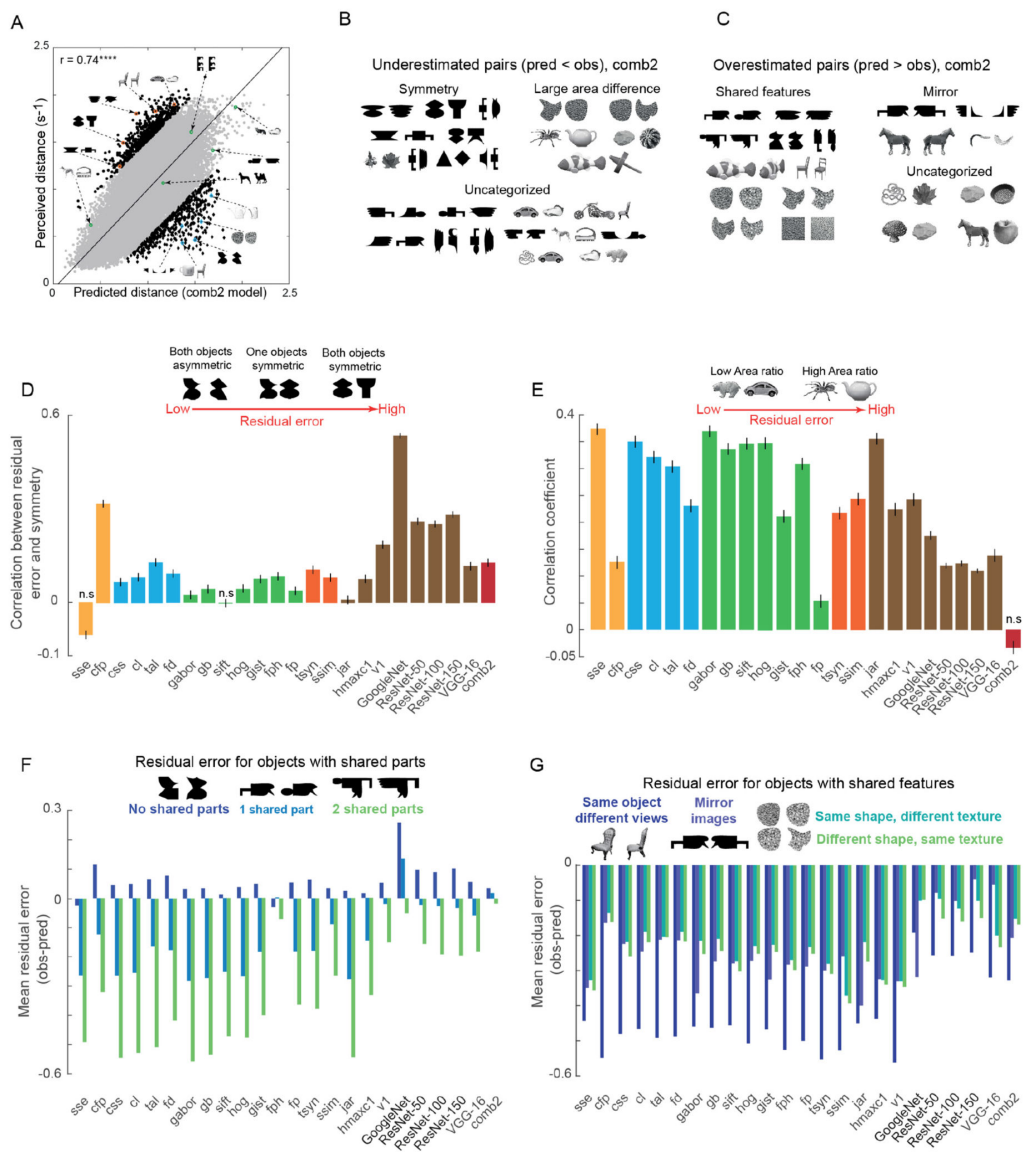
Model performance on perceptual data and residual patterns. (A) Correlation between predicted and observed distances for the best model (*comb2*) for all 26,675 pairs. Object pairs whose dissimilarity is underestimated by the model (residual error more than 1 standard deviation above the mean) are shown as filled black circles with example pairs highlighted in orange. Pairs whose dissimilarity is overestimated by the model (residual error less than 1 standard deviation below the mean) are shown as filled black diamonds with example pairs highlighted in blue. Pairs whose dissimilarity is explained by the model (residual error within 1 standard deviation of the mean) are shown as gray circles with example pairs highlighted in green. **** is p < 0.00005. (B) Examples of under-estimated pairs of objects; (C) Examples of overestimated pairs of objects; (D) Correlation between strength of symmetry and residual error across object pairs for each model. Error bars indicate bootstrap estimates of standard deviation (n = 10). All correlations are significant with p < 0.005 unless indicated by n.s (not significant); (E) Correlation between area ratio and residual error across object pairs for each model; (F) Average residual error across image pairs with zero, one or two shared parts; (G) Average residual error for object pairs related by view, mirror-reflection, shape and texture.

Does combining all models in some way produce even better fit to the data? To answer this, we quantified how the two combined models (*comb1* and *comb2*) fit to the perceptual data. It has to be noted here that all the tested models (including the combined models) have access to the entire dataset and cross-validated in the same way. We found that the comb1 model, in which features were concatenated before performing PCA, yielded a performance worse than even some individual models. We speculate that this may have been because concatenating many model features leads to correlated but irrelevant variations that are captured in the PCA. By contrast, the *comb2* model, in which the net distance is a weighted distance of all individual models, gave the best match to perceptual data (% variance explained = 68.1%; r = 0.74, p < 0.00005; Figure 2A). To identify the models that contributed the most and least to the *comb2* model, we inspected the weights associated with each model. VGG-16 and V1 model distances contributed the most, while Fourier Descriptor and Curvature Length model distances contributed the least.

#### 2.4.2 Systematic residual error patterns across all models

It is evident from the above analyses that even the best model doesn’t explain all the explainable variance in the data. To investigate this gap in greater detail, we calculated the residual error for each pair of objects as the signed difference between the observed distance and predicted distance. We then examined all image pairs whose residual error was one standard deviation away from model predictions. This revealed some systematic patterns. Image pairs whose dissimilarity was underestimated by the model (i.e. predicted < observed) frequently contained symmetric objects or pairs with objects having large area differences (Figure 2B). Image pairs whose dissimilarity was overestimated by the model (i.e. predicted > observed) contained objects that frequently shared features (Figure 2C). We found that these residual error patterns are not artefactual: using data from one half of the subjects to predict the other half revealed no such systematic errors.

To confirm that the above systematic error patterns were indeed present across all image pairs and in all models, we quantified these image properties and asked whether residual error increases systematically. These error patterns are investigated in greater detail in our previous study [34] and are only summarized here. First, we considered the specific case of symmetry. For each image pair, we calculated the average strength of symmetry in both images (see section 2.3.2) and asked whether this symmetry strength correlates with residual error for all models. A positive correlation would mean that as objects in a pair become more symmetric, model residual error increases – thereby confirming that symmetric objects are more distinctive in perception than in models. Indeed, all models including the best combined model (*comb2*) showed a significant positive correlation between strength of symmetry and residual error (Figure 2D). There were only two exceptions to this trend: the SSE and SIFT models, which showed no significant correlation. GoogleNet, though a better model at object recognition than VGG-16, doesn’t capture perceptual dissimilarities as well as other deep models. As a consequence, GoogleNet shows stronger residual error correlation. Further, the Coarse Footprint model captures differences in the overall shape of objects by blurring the internal details and hence, shows stronger residual error pattern as a result of underestimating dissimilarities (for both symmetric and asymmetric object pairs). In sum, almost all computational models underestimate the dissimilarity between symmetric objects.

Next, we quantified our observation that object pairs with large area differences are more distinct in perception. For each image pair, we computed the ratio of area of the larger object to area of the smaller object and correlated this ratio with the residual error for each computer vision model. We found that almost all models show significant positive correlation confirming that image pairs with large area differences show larger residual errors (Figure 2E). Here, the only exception was the *comb2* model (r = −0.03, p = 0.08).

Finally, we quantified our observation that dissimilarities between objects with shared parts are underestimated by computational models. To this end, we measured the average residual error for pairs of objects that shared two parts, one part or no part at all. We found that, for many models, the residual error was large and negative for objects sharing two parts, smaller but still negative for objects sharing one part and almost zero for objects with no shared parts (Figure 2F). Further, we found that the residual error was systematically negative for pairs that were constituted by two different views of the same object, pairs with mirror images of the same object, and pairs with either shared shape or texture (Figure 2G). Thus, objects with shared features or shared parts are more similar in perception compared to computational models.

#### 2.4.3 Generalization to novel experiments

**Fig. 3:**
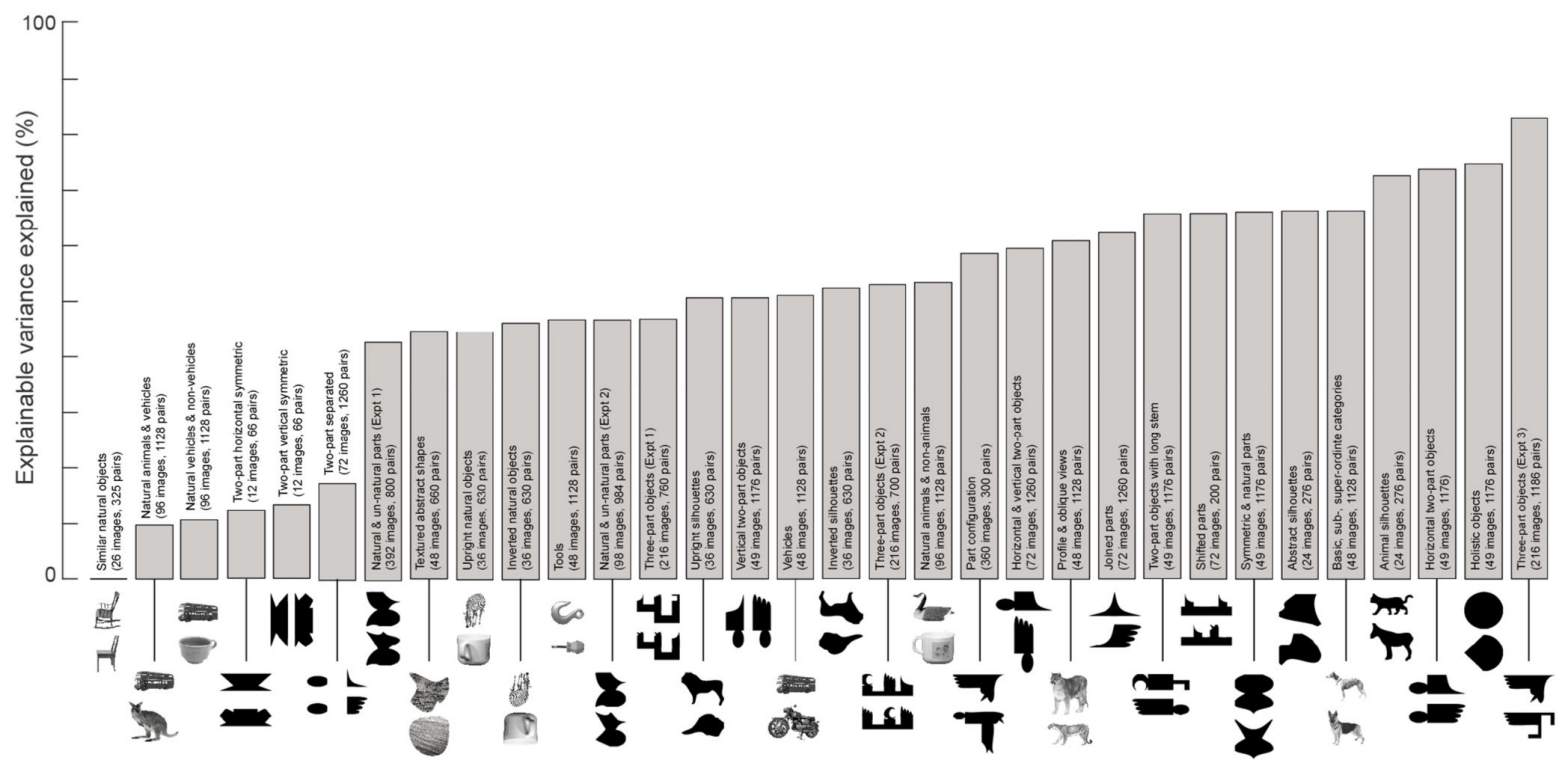
Generalization of the best model to novel experiments. Each bar represents the amount of variance explained by the best model (*comb2*) when it was trained on all other experiments and tested on that particular experiment. The text inside each bar summarizes the images and image pairs used, and the image centered below each bar depicts two example images from each experiment.

How robust are the above results to the set of object pairs chosen? The good cross-validation prediction of perceptual data by the best model (*comb2*) may not accurately represent its ability to generalize to novel images. This is because, the model is trained each time on 80% of the image pairs which may contain all the images in the dataset. To address this concern, we made use of the fact that our dataset of perceptual dissimilarities was compiled from 32 experiments with largely non-overlapping sets of images. We tested the performance of *comb2* model on each experiment after training it on all other experiments. This revealed a systematic trend – the model generalized poorly to experiments containing very similar natural objects, multiple views of various objects, and symmetric objects (Figure 3). Further, we set out to explore if these generalization trends hold even when the model was trained to predict data from the same experiment. We considered 16 experiments which had perceptual data for at least 1000 image pairs and trained the *comb2* model on 800 image pairs for each individual experiment with the testing done on the remaining 200 image pairs. We repeated this process 10 times to obtain an estimate of average variance explained. Here too, we saw similar trends as observed before with larger generalization errors for experiments containing similar natural images and symmetric objects.

## 3 Augmenting Cnns With Symmetry Features

In the previous section we described how computational models deviate systematically from human perception. In particular, one systematic bias is that symmetric objects are more distinct in perception compared to all computational models. If symmetry is represented differently in perception compared to computational models and in particular CNNs, then we reasoned that augmenting a state-of-the-art CNN with symmetry features would improve its performance.

### 3.1 CNN and dataset selection

We selected two CNNs – RCNN [51] and VGG-16 [3] which were trained on PASCAL VOC 2007/2012 and ImageNet dataset respectively. We used the MATLAB implementation of faster-RCNN that gave a mean average precision (mAP) of 59.9% on the PASCAL VOC 2007/2012 dataset. Similarly, we downloaded a pre-trained VGG-16 network which has a top-1 error of 24.4% on ImageNet Challenge 2014. To evaluate if augmenting with symmetry features improves the performance of the network on training images, we used the PASCAL VOC and ImageNet datasets. Specifically we used 17,125 images from 20 categories from the PASCAL VOC 2012 *trainval* set and 544,546 images from 1000 categories from the ImageNet training set (with ground-truth bounding box).

### 3.2 Symmetry feature extraction and augmentation

To extract symmetry features, we computed symmetry with respect to horizontal (*S_h_*) and vertical (*S_v_*) axis as explained in previous section (see section 2.3.2 and Equation 3). In addition, to account for variations in the orientation of symmetry axis, we computed symmetry score for 8 orientation axes uniformly sampled between 0° and 180°. All classifiers were trained using existing MATLAB functions (*fitcdiscr*) using 10-fold cross-validation.

#### 3.2.1 PASCAL-VOC dataset

We ran all of the PASCAL-VOC 2012 trainval images through the RCNN and collected the output detections (both bounding boxes and detection confidence). In all, we had 135,157 detections from 17,125 images (for a detection threshold of 0.2). We kept the detection threshold considerably low to get as many hits as possible. Each detection can either be a true detection or false alarm depending on the ground truth labels. We then collected hits and false alarms for each category and trained linear classifiers to segregate true from false detections. First, we trained a linear classifier on the RCNN detection confidence scores. Then, we trained a linear classifier on symmetry scores calculated using Equation 3. Finally, we trained a linear classifier on the combined representation of RCNN detection confidence score and the confidence score of the classifier trained on symmetry features.

#### 3.2.2 ImageNet dataset

We took 544,546 images spanning 1000 categories from the ImageNet dataset with ground-truth bounding box annotations and extracted activations from the penultimate fully connected layer of VGG-16. We calculated the symmetry scores for all images using Equation 3. We then trained linear classifiers to separate positive from negative examples. Positive examples were drawn from same category images based on the ground truth labels (*n* ≈ 500) and equal number of negative examples were drawn from images belonging to the rest of the categories. We trained linear classifiers on the activations extracted from the last fully connected layer of the VGG-16 network and on symmetry scores separately. We then trained another linear classifier on the confidence scores of the two classifiers. Finally, we tested these classifiers on the ImageNet validation set with 50 images in each class.

Although we used only a subset of the ImageNet dataset with ground-truth bounding box annotations and computed symmetry scores on pixels within the bounding box, we found similar gains in performance when symmetry scores were computed on the entire image. Thus, we are reporting the results of the latter case.

#### 3.2.3 Augmentation procedure

**Fig. 4:**
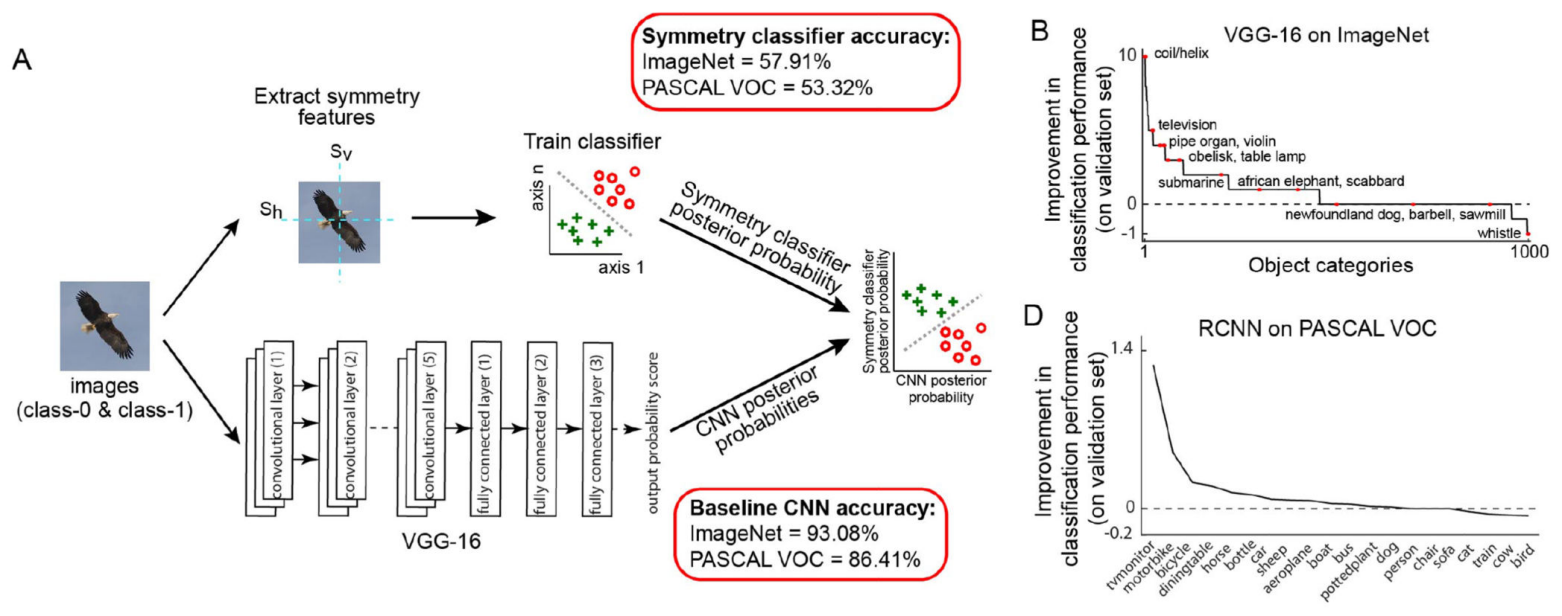
Augmenting CNNs with symmetry features. (A) Schematic of the pipeline used to augment symmetry information to CNN feature representation. Baseline CNN accuracy and symmetry classifier accuracy is shown for both ImageNet and PASCAL-VOC datasets; (B) Plot of improvement in classification performance of VGG-16 on augmenting with symmetry features computed on the validation set; (C) Similar plot as in (B) for RCNN on PASCAL-VOC dataset.

We used an augmentation procedure similar to the one used in [45]. Specifically, we first trained a binary linear classifier on the CNN representations (feature representation in the final fully connected layer of VGG-16 for ImageNet and RCNN detection confidence scores for PASCAL-VOC) and obtained posterior probability scores for both positive and negative examples. We then trained another binary linear classifier on symmetry features and obtained another set of posterior probability scores for both positive and negative examples. Finally, we trained a third binary linear classifier on the set of posterior probability scores computed from the first two classifiers to obtain predicted class labels. This augmentation pipeline is summarised in Figure 4A.

### 3.3 Results

The pipeline used to augment convolutional neural networks with symmetry features is summarized in Figure 4A and described in detail in the previous section. All three classifiers in the augmentation procedure were tested on an independent held-out set of images. Thus, if symmetry features are already learned by CNNs, then this procedure should not improve cross-validated detection accuracy. However, this was not the case. We observed significant gains in performance using VGG-16 on ImageNet validation set (average improvement: 0.82% across 1000 categories; Figure 4B). In fact, this improvement in classification accuracy was significant as assessed through statistical testing (median accuracy: 94% and 95% for VGG-16 before and after symmetry feature augmentation respectively; *p* < 0.000005 for a ranksum test on classification accuracies across 1000 categories of ImageNet validation set). Many categories showed an improvement when the VGG-16 scores were augmented with symmetry scores. Interestingly, 101 categories showed improvements of 3% or more with ‘coil, helix’ category showing improvements as high as 10%. Symmetry features by themselves yielded above-chance classification (average classification accuracy = 58% compared to chance accuracy = 50%).

This improvement in classification was not specific to the VGG-16 on the ImageNet dataset. On the PASCAL VOC 2012 trainval images, the classification performance of the RCNN improved upon including symmetry features (average improvement = 0.13% across all 20 categories; Figure 4C). Some categories showed improvements greater than 0.5% (improvement in classification accuracy: 1.3% for tv-monitor and 0.55% for motorbike). Here too, symmetry features by themselves yielded above-chance classification (average classification accuracy = 53.32% with chance accuracy = 50%). Thus, augmenting CNNs with symmetry features leads to significant improvements in performance.

The smaller gains in classification accuracy after augmentation can be due to two reasons. First, it could be a reason intrinsic to symmetry itself. Symmetry as a property can never perfectly discriminate object identity because it does not contain shape information. Second, it could be because our measure of symmetry is not perfect. The Im-ageNet dataset does not contain objects segmented from the background, so our symmetry scores may be corrupted by background pixels. The symmetry score may also be corrupted by image skew due to 3D rotations, natural shading variations across the image or by occlusion. The fact that we obtained an accuracy improvement even with our rudimentary measure of symmetry suggests that more sophisticated measures would lead to even better improvements.

We next asked why symmetry feature augmentation showed smaller gains on PASCAL VOC compared to Im-ageNet. One reason could be that images in PASCAL VOC dataset are less symmetric compared to images in ImageNet. Indeed, we found that ImageNet has a larger range of symmetry scores across categories compared to PASCAL VOC and the average symmetry score for each category significantly differed from a common mean for both datasets (p < 0.00005, for Kruskal-Wallis test on symmetry scores with category labels as factor). Further, we found that ImageNet has more symmetric images than PASCAL VOC (average symmetry score, mean ± std: 0.78 ± 0.04 and 0.73 ± 0.03 for ImageNet and PASCAL VOC dataset respectively, p < 0.00005 for rank-sum test on category-wise average symmetry scores). Thus, augmenting with symmetry features leads to smaller gains on PASCAL VOC compared to ImageNet dataset. In general, the augmentation procedure can lead to significant gains in performance depending on the biases present in the dataset.

Why does augmenting with symmetry improve CNN accuracy? We examined two possibilities. First, we asked whether augmenting with symmetry improved categories on which the VGG-16 network performed badly. This was indeed the case: improvements in accuracy were negatively correlated with VGG-16 classification accuracy (correlation between improvement in classification accuracy and VGG-16 accuracy: r = −0.50, p < 0.00005 across 1000 categories in ImageNet). Second, we surmised that highly symmetric or highly asymmetric objects would experience the greatest increases in accuracy. Indeed, objects such as coil, dragonfly, solar dish, park bench, and flagpole showed the largest improvement. To quantify this pattern, we asked whether the average strength of symmetry for each object category (calculated as the average score across all positive examples) was correlated with performance improvement. This revealed a positive correlation (r = 0.28, p < 0.000005 across 1000 categories in ImageNet), suggesting that, as expected, symmetric objects benefited the most from augmenting CNNs with symmetry features.

Finally, we note that there are other ways of incorporating symmetry features into the CNN, which may well yield better improvements in performance. We explored one appealing alternative: We concatenated the activations of the final fully connected layer with symmetry features (after z-scoring each feature across images) and used this augmented feature vector (with 1000 features from VGG-16 and 8 symmetry score features) to learn a new object classifier. We evaluated the performance of this classifier by training binary linear classifiers on equal numbers of positive and negative examples in each category using 5-fold crossvalidation. Interestingly, this produced no improvement in accuracy (average improvement across 1000 categories: −0.007 ± 0.22%). Thus, augmenting classifiers produces better performance than augmenting features themselves. A similar result has been reported previously in comparing early versus late fusion of features [52].

## 4 Understanding Why Cnns Underestimate Symmetry

So far we have shown that machine vision algorithms show systematic biases from human vision, and that fixing one of these biases by augmenting CNNs with symmetry features leads to significant improvements in performance. These results show that symmetric objects are more distinct in perception compared to CNNs but do not explain why this is so.

**Fig. 5:**
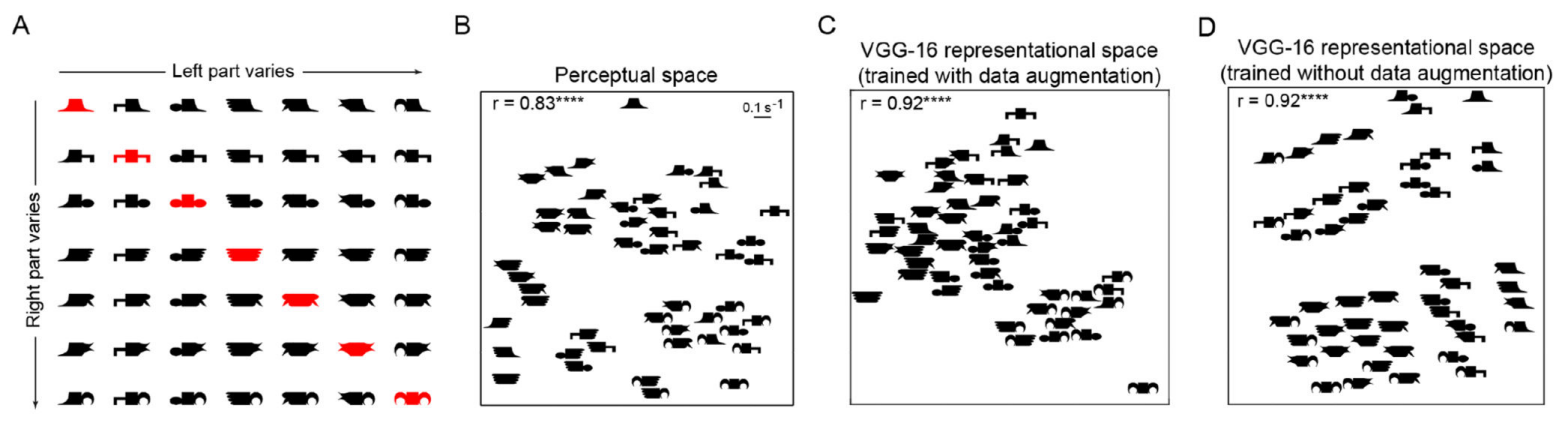
Representation of symmetric and asymmetric objects in perception and CNNs. (A) Set of 49 two-part objects used to explore representation of symmetric objects in both perception and CNNs. Symmetric objects are highlighted in *red*. (B) Visualization of perceptual space using Multidimensional Scaling (MDS). *r* indicates the Pearson’s correlation coefficient between perceived distances and distances in the 2D plot, **** is p < 0.00005 (C) Similar plot as in (B) for the penultimate fully connected layer of VGG-16. (D) Similar plot as in (C) for VGG-16 trained without data augmentation.

To address this issue, we systematically analyzed object representations in the penultimate fully-connected layer of VGG-16 for a subset of objects in the dataset. We chose the penultimate fully-connected layer activations for this analysis as this can be considered the last representational layer whose output is used for classification. The subset of objects used for the analysis, shown in Figure 5A, consists of 7 arbitrary parts combined in all possible ways to create a total of 49 objects. We measured visual search dissimilarities as well as VGG-16 feature distances for all possible pairs of these 49 objects (*n* = ^49^ C_2_ = 1,176 pairs).

To visualize these representations, we used multidimensional scaling. The resulting plot for perceptual dissimilarities is shown in Figure 5B – in this plot, nearby objects represent hard visual searches. It can be seen that objects that share parts are closer together, and that symmetric objects are far apart. The resulting plot for the VGG-16 representation is shown in Figure 5C – in this plot, nearby objects are those that evoked similar activation across the penultimate fully connected layer. It can be seen that the VGG-16 representation shares many features with the perceptual representation: objects that share parts are again closer to each other, and symmetric objects are further apart in general. There was a strong positive correlation between pairwise object distances of the VGG-16 representation with perception (r = 0.68, p < 0.00005).

**Fig. 6:**
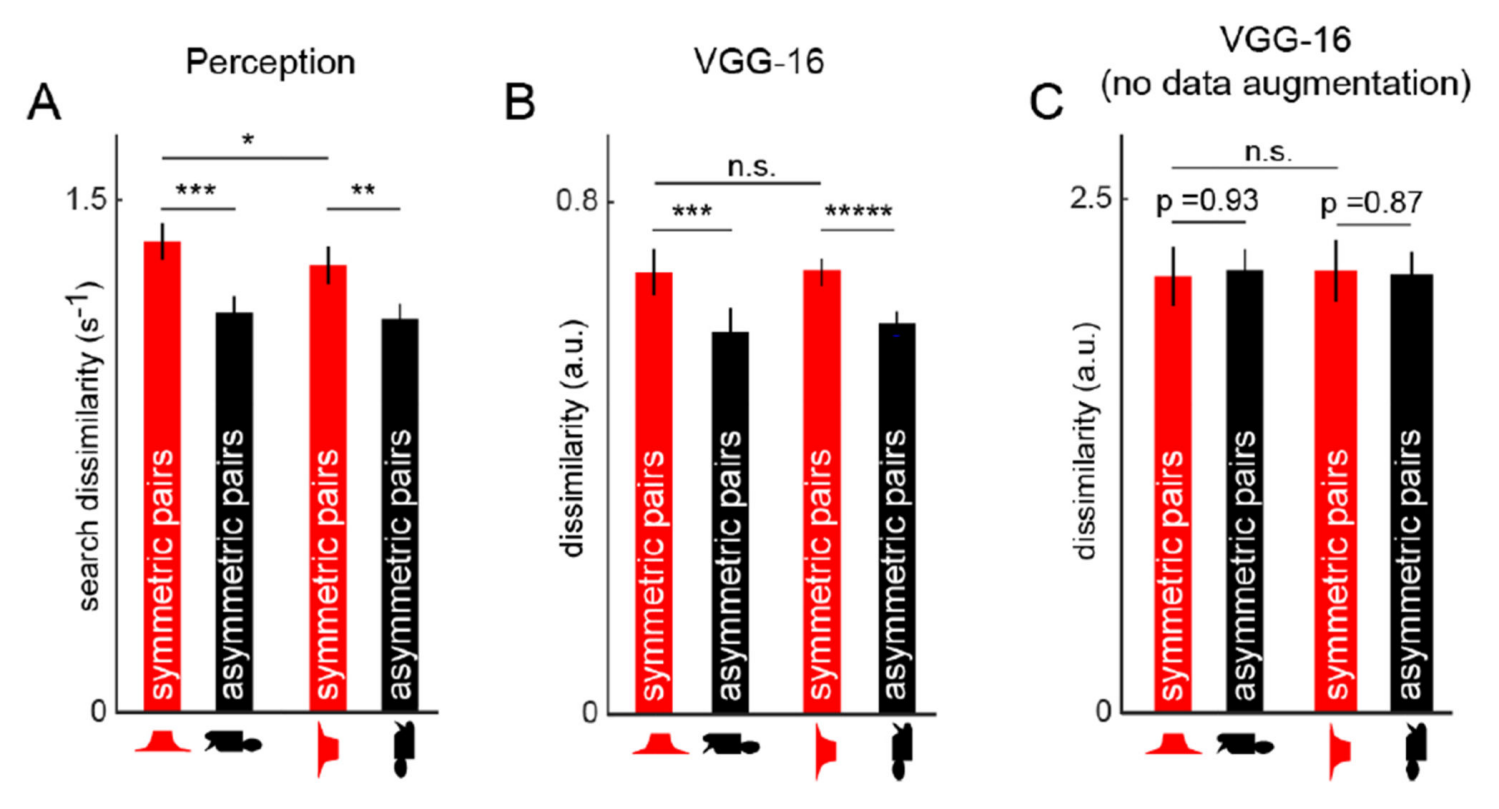
Symmetry advantage in perception and CNNs. (A) Perceptual dissimilarity in humans for both horizontal and vertical symmetric and asymmetric object pairs. Asterisks represent statistical significance of comparisons: * is p < 0.05, ** is p < 0.005 and *** is p < 0.0005. (B) Similar plot as in (A) for the penultimate fully connected layer of VGG-16. n.s. is not significant and ***** is p < 0.000005. (C) Similar plot as in (A) for the penultimate fully connected layer of a VGG-16 network trained without data augmentation.

To quantify the observation that symmetric objects are far apart, we compared the distance between pairs of symmetric objects (^7^ *C*_2_ = 21 pairs) with distances between pairs of objects differing in two parts (pairs of the form AB-CD; n = 420 pairs). This revealed a statistically significant difference (mean ± std distance: 1.36 ± 0.24 *s* ^-1^ for symmetric pairs, and 1.16 ± 0.21 *s* ^-1^ for asymmetric pairs, p < 0.0005, rank-sum test on distances; Figure 6A). This was true for the VGG-16 penultimate fully-connected layer (mean ± std of distance: 0.74 ± 0.17 for symmetric pairs and, 0.61 ± 0.09 for asymmetric pairs, p < 0.005, rank-sum test on distances; Figure 6B). We also confirmed this trend for vertically-oriented objects created by rotating the objects shown in Figure 5A counter-clockwise by 90°. That is, symmetric object pairs were statistically more dissimilar than asymmetric object pairs both in perception (mean ± std distance: 1.31 ± 0.26 *s* ^-1^ for symmetric pairs, and 1.15 ± 0.2 *s* ^-1^ for asymmetric pairs, p < 0.005, rank-sum test on distances; Figure 6A) and the penultimate fully-connected layer of VGG-16 (mean ± std of distance: 0.74 ± 0.17 for symmetric pairs and, 0.61 ± 0.09 for asymmetric pairs, p < 0.005, rank-sum test on distances; Figure 6B). Interestingly, we found that horizontal symmetric objects were significantly more dissimilar than vertical symmetric objects in perception (p < 0.05 for a ranksum test on dissimilarities; Figure 6A) but not in VGG-16 (p = 0.07 for a rank-sum test on dissimilarities; Figure 6B). This difference between horizontal and vertical symmetry is very well established in literature where symmetry about the vertical axis is detected faster than symmetry about the horizontal axis [22], [53], which in turn is believed to be related to the distinctiveness of these objects [22].

Thus, symmetric objects are distinctive both in perception and in VGG-16.

### 4.1 Are symmetric objects special in CNNs trained without image flipping?

The fact that symmetric objects are more distinctive compared to asymmetric objects in VGG-16 could be due to the nature of its training, where each image and its mirror-reflected version are used for robustness. Alternatively it could be present due to mirror images present in the dataset itself, due to the presence of bilaterally symmetric objects that produce mirror images across views. Therefore we wondered whether the symmetry advantage would still be present if the VGG-16 network was trained without mirror-flip data augmentation.

To investigate this issue, we trained a VGG-16 network from scratch on the ImageNet training dataset containing ∼1.2 million images from 1000 object categories to perform object classification. The network was trained for 100 epochs with a batch-size of 20 using PyTorch framework on NVIDIA TITAN-X/1080i GPUs. The generalization capability of the model was tested on the ImageNet validation set which has 50,000 images from the same 1000 object categories as in the training set. The VGG-16 network trained without data augmentation showed good generalization (average ± std of top-1 accuracy: 56% ± 19% and top-5 accuracy: 80% ± 14% over 1000 object categories). By contrast, the VGG-16 network trained with augmentation has better generalization (average top-1 accuracy: 75.6% and top-5 accuracy: 92.9%; [3]).

Next we analyzed symmetric and asymmetric object representations in the VGG-16 network trained without data augmentation using the same set of two-part objects as before (Figure 5A). To visualize the underlying representation, we used multidimensional scaling as before. In the resulting plot (Figure 5D), it can be seen that objects that share the left part cluster together separately from objects that share the right part, and there is no apparent advantage of symmetric objects. Indeed, distances between symmetric objects were no greater than between other asymmetric objects (mean ± std of distance: 1.92 ± 0.45 and 1.96 ± 0.3 for 21 pairs of symmetric and 420 pairs of asymmetric objects respectively; p = 0.93 for a rank-sum test on distances; Figure 6C). This trend remained true even for vertical objects (mean ± std of distance: 1.95 ± 0.49 and 1.99 ± 0.31 for 21 pairs of symmetric and 420 pairs of asymmetric objects respectively; p = 0.87 for a rank-sum test on distances; Figure 6C). The regularity in arrangement of objects as shown in Figure 5D might arise from position-dependent shape tuning in the network trained without mirror-flipped images.

We conclude that CNNs trained without mirror-flip data augmentation do not show the symmetry advantage.

### 4.2 Understanding the CNN-perception difference

The results above show that the standard VGG-16 CNN (trained with data augmentation) shows a symmetry advantage just like in perception, albeit lower in magnitude. This difference may partially explain why augmenting with symmetry improved its performance. A further reason why augmenting worked could be that units that contribute more to object classification show a weaker symmetry advantage.

#### 4.2.1 Identifying units important for classification

To address this issue, we calculated a measure of overall contribution towards classification for each unit [54]. We randomly selected 20 images from the ImageNet validation set from different classes that were classified correctly by the VGG-16 network. We computed the importance of each unit *n_i_* in the penultimate fully-connected layer as follows. First, we removed the contribution of unit *n_i_* towards classification by zeroing the weights going out from *n_i_* to all units in the final fully-connected layer. We then passed all 20 images through this modified VGG-16 network and also the original VGG-16 network and computed the change in output class probabilities. Finally, we defined the importance of *n_i_* as

(4) $$\delta \left(n_{i}\right)=\frac{1}{20}\overset{20}{\underset{j=1}{\sum }}|(p_{o}\left(c_{j}\right)-p_{m}\left(c_{j}\right)|$$

where δ(*n_i_*) is the importance of unit *n_i_*, *p_o_*(*c_j_*) is the output probability for image *j* corresponding to the true class *c_j_* for the original VGG-16 network, and *p_m_*(*c_j_*) is the corresponding class probability for the *modified* VGG-16 network.

#### 4.2.2 Symmetry advantage in units important for classification

Next we asked whether the units with high importance show a weaker symmetry advantage. To this end we calculated a symmetry modulation index (SMI) as

(5) $$SMI=\frac{d_{sym}-d_{asym}}{d_{sym}+d_{asym}}$$

where *d_sym_* and *d_asym_* are the average distances for symmetric and asymmetric object pairs respectively. We estimated the average symmetry modulation index by bootstrap i.e. by randomly sampling with replacement 21 symmetric object pairs and 420 asymmetric object pairs. We repeated this procedure to get 10,000 bootstrap estimates of symmetry modulation index each for perception, all units in the penultimate fully-connected layer of VGG-16, top-100 and bottom-100 units in the penultimate fully-connected layer of VGG-16, and all units in the penultimate fully-connected layer of VGG-16 trained without data augmentation.

**Fig. 7:**
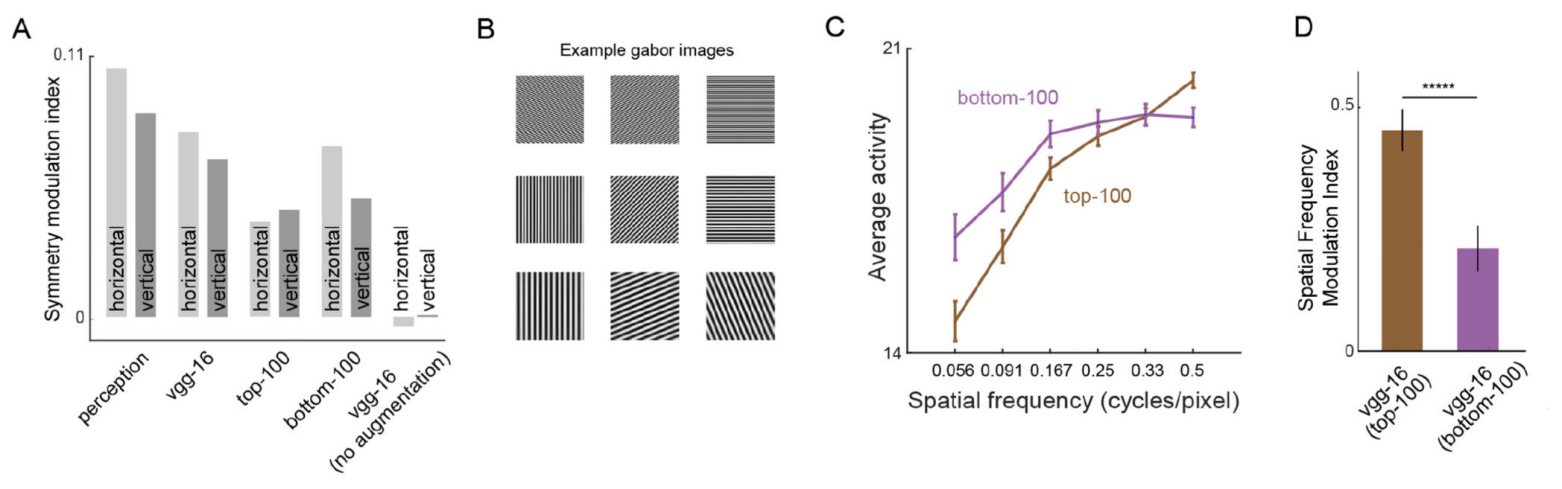
Symmetry advantage in units important for classification. (A) Symmetry modulation index (Eq. 5) for horizontal and vertical objects. (B) Example gabor images used for the spatial frequency analysis (C) Average activity evoked by Gabors of varying spatial frequency for top-100 and bottom-100 units in the penultimate fully-connected layer of VGG-16. Error bars indicate s.e.m. across units; (D) Spatial frequency modulation index (Eq. 6) for top-100 and bottom-100 units. Error bars indicate s.e.m. ***** is p < 0.000005.

The average SMI for both horizontal and vertical objects are shown in Figure 7A. The symmetry modulation index was highest for perception, followed by VGG-16, bottom-100 units, top-100 units and VGG-16 trained without data augmentation. As hypothesized, SMI for the top-100 units were smaller compared to the bottom-100 units for both horizontal and vertical objects indicating that units important for classification show weaker symmetry advantage.

#### 4.2.3 Feature analysis of units important for classification

The above result shows that the top-100 units in the penultimate fully connected layers are systematically different from the remaining units in terms of representation of symmetry. Are they selective for different features compared to the rest of the units? We investigated this issue by comparing top-100 and bottom-100 units in the VGG-16 network using a widely used feature analysis technique from neuroscience, as detailed below.

We wondered whether the top-100 and bottom-100 units differed in their spectral power preferences. To assess this possibility, we created Gabor images (see some examples in Figure 7B) with 8 orientations (uniformly sampled from 0 to 180 degrees) and 6 spatial frequencies (0.06, 0.09, 0.17, 0.25, 0.33 and 0.5 cycles/pixel) and obtained CNN unit activations to these images from the top-100 and bottom-100 units. For each unit, we computed its average activation for each spatial frequency by averaging its activation across orientations. The average behaviour of the top-100 and bottom-100 units is shown in Figure 7C. The average activity of the top-100 units was relatively low for low spatial frequencies and increased for high spatial frequencies. In contrast, the bottom-100 units showed a steady response to high spatial frequencies. To quantify the relative preference for high over low spatial frequencies for each unit *n_i_*, we calculated a spatial frequency modulation index as

(6) $$MI\left(n_{i}\right)=\frac{A_{hsf}-A_{lsf}}{A_{hsf}+A_{lsf}}$$

where *A_hsf_* is the average activation for unit *n_i_* computed for high spatial frequency images (0.25, 0.33 and 0.5 cycles/pixel) and *A_lsf_* is the average activation for unit *n_i_* computed for low spatial frequency images (0.06, 0.09 and 0.17 cycles/pixel). The average spatial frequency modulation for top-100 units was significantly larger compared to the bottom-100 units (Figure 7D; p < 0.0005 for a ranksum test on modulation indices for top-100 and bottom-100 units). Thus, VGG-16 units important for classification respond more to high spatial frequencies compared to low spatial frequencies, indicating that they may be tuned to spatially local features. We surmise that this could be the reason for their weaker symmetry advantage.

## 5 Discussion

Here we have compared perceptual dissimilarity in humans with a variety of computational models. Our main finding is that all machine algorithms tested show systematic biases from human perception. Furthermore, fixing one of these biases (symmetry) can improve CNN performance. We have further shown that CNNs show a weak advantage for symmetry particularly among the units important for classification. In a recent study, we showed that the advantage for symmetry in perception arises due to similar part selectivity on either side of an object [22]. We therefore propose that consistent part selectivity could be imposed as a constraint during learning, and that doing so will improve performance.

Our improvements in performance may have been small due to noisy estimates of symmetry features. Recent advances in geometry processing using classical methods as well as deep learning have led to better symmetry detectors both on 3D models of objects [25], [29], [31] and 2D objects embedded in natural scenes [26], [30]. Further, there have been efforts to reduce the sample complexity of deep neural networks by designing convolutional filters that capture various symmetries in the training data [27], [28]. Although these are significant advances in symmetry detection, they haven’t been tested on large-scale datasets in the context of object recognition tasks. We speculate that combining our insights about human perception with better symmetry measures will lead to larger improvements in performance, particularly on real-world vision tasks.

Finally, we note that symmetry is not the only systematic difference we have observed between human perception and machine vision. Objects with large area differences, mirror images and objects with shared features all show systematic deviations. Augmenting CNNs with these properties is less straightforward but one possibility is to use perceptual data as an additional constraint during learning [55].
